# Medical error professionals’ perspectives on Inter-system Medical Error Discovery (IMED): Consensus, divergence, and uncertainty

**DOI:** 10.1097/MD.0000000000021425

**Published:** 2020-07-31

**Authors:** Jacquelyn Miller, C. Ann Vitous, Richard C. Boothman, Lesly A. Dossett

**Affiliations:** a Center for Bioethics and Social Sciences in Medicine (CBSSM); b Center for Healthcare Outcomes and Policy (CHOP); c Department of Surgery, University of Michigan, Ann Arbor, MI, USA.

**Keywords:** clinical ethics, disclosure, ethics, feedback, guidelines, medical error, patient safety, reporting

## Abstract

Best practices for how to respond are unclear when a medical error is discovered in a different system (inter-system medical error discovery or IMED). This qualitative study explored medical error professionals’ views on disclosure, feedback, and reporting in these scenarios.

We conducted semi-structured telephone interviews from January to September 2018 with 15 medical error professionals from 5 regions of the United States. Interview guides addressed perspectives on best practice, minimum obligations, and mediating factors with respect to IMED. Each transcript was coded independently by two investigators. Analysis followed the inductive approach of interpretive description.

Medical error professionals expressed diverse views about minimum obligations and best practices for physicians when responding to IMED events. All cited practical barriers to disclosure, feedback, and reporting in these scenarios. There was general consensus that clear-cut, harmful errors should be disclosed to patients, and most advised investigation and feedback prior to disclosure. Respondents diverged in recommended best practices and thresholds for taking action. All noted the lack of guidance specific to IMED scenarios but differed in how they would extrapolate from more general guidance.

While medical error professionals expressed consensus regarding obligations to disclose obvious errors, they differed on particulars. Guidelines or an algorithm could be very useful. Efforts to develop clear guidelines for IMED must take into account these factors, as well as practical and political challenges to communication about errors discovered across systems.

## Abstract 2

Supplemental Digital Content is available in the text

## 1 Introduction

To avoid future medical errors, improve transparency, and mitigate malpractice risks, organizations have adopted strategies for disclosure and emphasized quality improvement through peer review.^[1]^ This focus has led to general consensus on how providers and hospitals should respond to their own errors:

1. disclose errors to patients and families,
2. report incidents and utilize peer review for a systems-based approach to preventing errors, and
3. offer financial or other compensation to reduce malpractice risks and provide resolution for patients and families.^[2]^

In a fragmented healthcare system, providers may discover other providers’ errors that are previously unknown to the patient and responsible provider, a scenario we call inter-system medical error discovery (IMED).^[3,4]^ Although data are limited, evidence suggests providers are reluctant to disclose others’ errors^[5,6]^ or to provide feedback to other providers particularly when they work in other health systems, citing barriers including medico-legal implications, concern for referral base, lack of time, and general discomfort providing negative feedback.^[7,8]^ Best practices for how providers or hospitals should respond when an error is discovered in a different system are unclear. A comprehensive analysis of ethical and professional guidelines in healthcare found narrow or ambiguous terminology and guidance, often with conflicting signals about prioritizing transparency versus avoiding criticism of fellow practitioners.^[9]^

The limited literature that does provide guidance on IMED scenarios generally endorses feedback between providers and is conflicting regarding disclosure to patients.^[10–18]^ Typically these have taken the form of opinions or case commentaries that do not fully confront the complexities of IMED scenarios. Some authors focused on feedback^[10,11]^ and others mainly on intra-institutional scenarios.^[12,13]^ Those who addressed IMED disclosure differed on who should perform disclosure (the responsible provider, discovering provider, or a medical director)^[12,14,15]^ or whether it should take place at all, often citing the lack of clear standards from professional societies.^[13,16–18]^

Professionals such as ethicists, risk managers, patient safety and quality improvement experts, and medical attorneys regularly encounter medical errors and are important stakeholders in efforts to develop clear guidelines for IMED. In this exploratory qualitative study, we sought to investigate their views on best practices and minimum obligations with respect to disclosure, feedback, and reporting of medical errors discovered across systems. Our aim was to determine the degree of consensus or divergence on best practices and minimum obligations and to elicit the mediating factors considered most relevant by medical error professionals. Asking these experts to unpack the challenges of IMED scenarios in detail will enable more practicable guidance to be developed in the future.

## 2 Methods

We conducted semi-structured interviews from January to September of 2018 with professionals in the United States who regularly encounter medical errors. The Institutional Review Board of the University of Michigan Medical School approved the study. This study is reported according to the Consolidated Criteria for Reporting Qualitative Research (see Appendix).^[19]^

Due to their special expertise, participants were recruited through professional contacts of the study team, websites of risk manager professional associations, and respondent-driven sampling.^[20]^ Purposive sampling was used to recruit from varied disciplines in order to elicit perspectives of multiple stakeholder groups including ethicists, risk managers, patient safety and quality improvement experts, and medical attorneys. After regional variation was identified as a salient factor in early interviews, participants were also recruited from different regions.

A semi-structured interview guide was adapted from previous studies^[6,8,21]^ and tailored to the expertise of the professional (Supplementary Material A). We first asked participants to describe their professional background. Then we provided the Institute of Medicine definition of medical error and elicited a case of inter-system medical error discovery in the participant's own experience. For each topic of disclosure, feedback, and reporting, questions elicited views about best practices, minimum obligations, and salient factors to consider in determining best practice.

Verbal consent was obtained prior to each interview. One researcher (JM) conducted all interviews, which were audio-recorded, transcribed verbatim, and summarized in real time to highlight points of similarity or contrast with previous interviews. The team reviewed these summaries periodically as a means of preliminary analysis to inform the questions in subsequent interviews (constant comparative method).^[22]^

After 15 interviews were conducted, 2 researchers (JM and CAV) with training in social sciences and qualitative methods independently coded each one, using MAXQDA Plus (VERBI Software 2018, Berlin, Germany) to facilitate analysis. They met after coding batches of 2 to 4 interviews and arrived at consensus through discussion, while collaboratively developing a codebook (Supplementary Material B). Structural and descriptive coding were used to index the topics of the interview. Most codes were anticipated *a priori* based on previous research and the interview summaries, while others were developed inductively. The team assessed that the 15 interviews had adequately identified salient factors and highlighted some convergent and numerous divergent views. Therefore, the data had sufficient information power in relation to the study aim,^[22,23]^ and it was determined that no further interviews were required.

We analyzed data following the methodology of interpretive description.^[22,24]^ This method uses “inductive analytic approaches designed to create ways of understanding clinical phenomena that yield applications implications” and “illuminate their characteristics, patterns, and structure.”^[24]^ In addition to writing and reviewing interview summaries and coding transcripts, we abstracted data from each interview to a framework matrix to facilitate case comparison.^[25]^ We explored consistent and surprising patterns and examined these themes across cases and across topics of disclosure, reporting, and feedback. We further examined contrasting and outlying cases.

## 3 Results

Participant characteristics are provided in Table 1. Notably, 9 participants had experience in 2 or 3 relevant fields. Only 2 participants reported having previously considered the topic in depth; all were able to provide an example from experience.

**Table 1:**
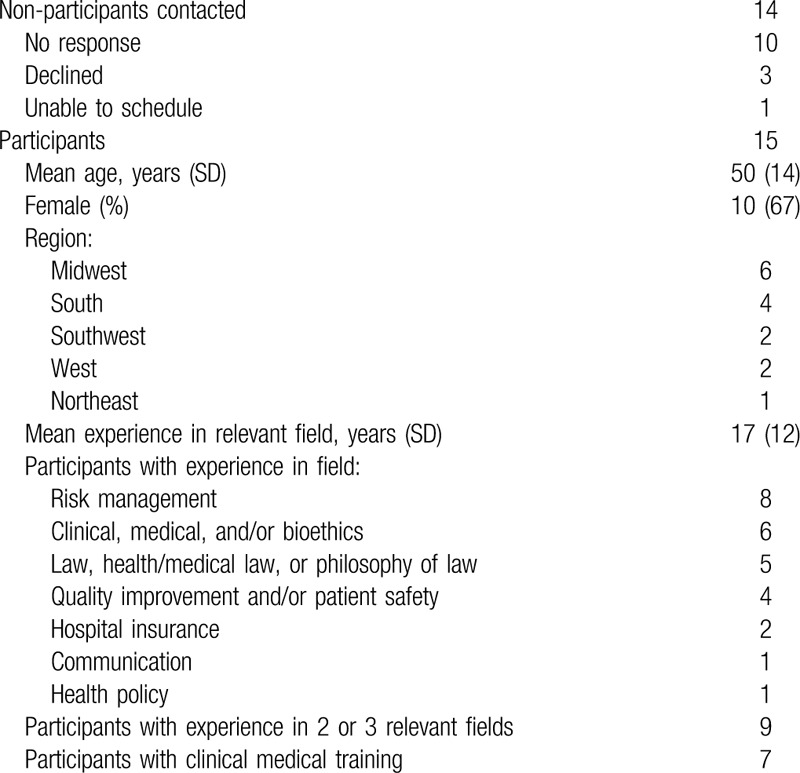
Participant characteristics and non-participation.

### 3.1 Disclosure to patients

Respondents’ views on minimum obligations for disclosure of IMED to patients hinged primarily on factors of uncertainty and severity. Recommendations for best practices emphasized the sequence of feedback and disclosure, the manner of disclosure, and the need for ancillary institutional supports.

#### 3.1.1 Clear-cut errors

To assess baseline consensus about inter-system medical error discovery, participants were asked to assume the errors in question are clear-cut and unambiguous. In such cases, 12 participants described best practice as full, timely transparency in disclosure to patients. Four volunteered that the standards for disclosure should be no different than standards for errors within one's own health system. Of the dissenters from this consensus, 1 recommended providing only facts relevant to the patient's future care and 2 asserted that errors discovered across systems are rarely clear-cut and therefore disclosure should not be mandatory (see Table 2 for illustrative quotes).

**Table 2:**
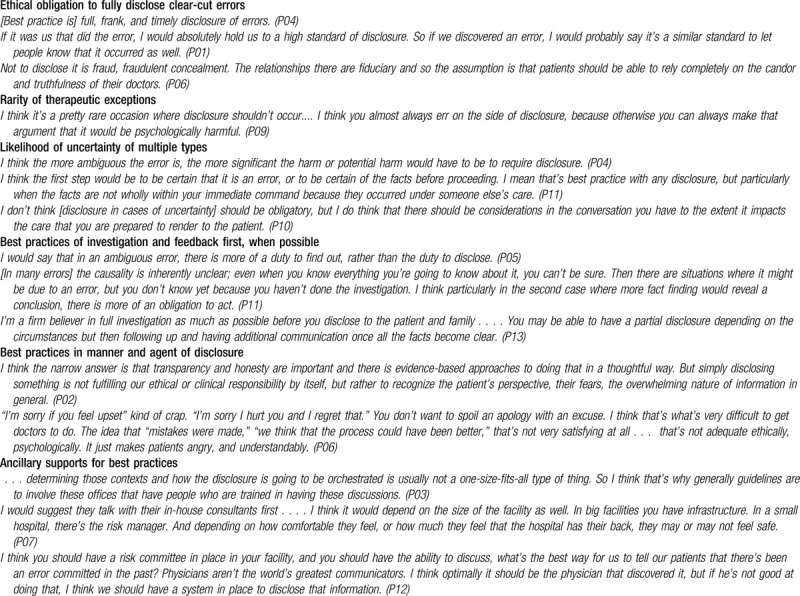
Best practices and minimum obligations for disclosure of Inter-system Medical Error Discovery to patients.

#### 3.1.2 Uncertainty about errors

In cases of uncertainty about whether an error occurred or who was responsible for it, the broad agreement on minimum standards broke down to a range of views on disclosure obligations. Seven respondents recommended a best practice of investigating and seeking clarification with the responsible provider, a form of feedback prior to disclosure. Several described a strong obligation for investigation before disclosure to avoid confusion, and especially if it could result in a conclusion about whether an error indeed occurred. In contrast, if the uncertainty is only about who is responsible, one respondent stated that the obligation to disclose remains. Some described an obligation to acknowledge a potential error while at the same time “declaring” the uncertainty. Others maintained that there is much less obligation to disclose in cases of uncertainty, the obligation to disclose can only be a fact-based case-by-case decision, disclosure is only obligated if necessary to explain future care, or only known facts should be stated without using the word “error” (Table 2).

#### 3.1.3 Severity of error

Error severity was the second-most frequently discussed factor in relation to minimum obligations for disclosure with respondents expressing divergent views. Some believed it ethically obligatory to disclose even harmless errors, others proposed an obligation to disclose any potential impact to patient health or finances, while others saw a minimum obligation to disclose only in cases of patient harm. Respondents discussed the interaction of uncertainty with these severity thresholds. One stipulated that the obligation to disclose increases with the severity of the error while decreasing with uncertainty and another stated that cases with uncertainty and no harm require a case-by-case determination.

#### 3.1.4 Investigation and feedback prior to disclosure

Eleven respondents recommended a best practice of communication with the responsible provider or the provider's health system prior to disclosure whenever possible. They cited multiple functions of this approach:

1. in cases of uncertainty, investigation and clarification to determine whether a medical error occurred and who was responsible;
2. even with apparent certainty, investigation of contextual factors surrounding the error that may be relevant to disclosure;
3. allowing or encouraging the responsible provider to perform the disclosure; and
4. ensuring that everyone involved in disclosure provides consistent and accurate information, avoiding unnecessary confusion for the patient.

Some respondents noted that the time required to perform such an investigation across facilities is one of the main practical obstacles to disclosure in this context.

According to several respondents, if the responsible provider proves unwilling to perform the disclosure, then the discovering provider is obligated to disclose or ensure disclosure happens. An exception to the best practice of communicating with the responsible provider first may be situations where disclosure is urgent for medical, practical, or regulatory reasons; in such cases, several said that best practice would be timely disclosure followed by investigation and later follow-up with patients when more facts are known. The emphasis on investigation and feedback first highlights that most respondents advised withholding final determination of an error until after consultation with the responsible provider (Table 2).

#### 3.1.5 Manner of disclosure

Best practices unique to IMED scenarios included encouraging the responsible provider to disclose the error (perhaps jointly with the discovering provider). Alternatively, some advised that the discovering provider give a basic disclosure (including facts and sufficient information for the patient to evaluate quality of care from the responsible provider) and then refer the patient to the responsible provider for a full discussion. Four respondents emphasized avoiding any negativity or blame about the responsible provider (Table 2).

#### 3.1.6 Ancillary supports and processes

Despite strong views that error disclosure is ethically obligated, none of the respondents were aware of any published guidelines specific to IMED scenarios. Several suggested adding these scenarios to existing guidelines on disclosure and reporting or developing an algorithm to guide discovering providers on how to respond to various error types.

Others shared the perspective that the complexity and variability of contextual factors in IMED would make guidelines less useful and instead advised consulting with the discovering hospital's error resolution teams to help plan, guide, or lead the disclosure conversations. Many recommended “just-in-time”^[26]^ coaching or proactive training in communications skills ranging up to formal, mandatory disclosure training. However, respondents also noted that smaller facilities do not have these infrastructural supports. One respondent emphasized that no matter the trainings available, institutional leadership and role modeling take primacy (Table 2).

### 3.2 Feedback to responsible providers

As described above, when asked about disclosure to patients many medical error professionals recommended communication with the responsible provider. Such communication necessitates feedback to the responsible provider, but respondents often framed this in terms of advantages for the disclosure process rather than for the sake of feedback itself.

#### 3.2.1 Obligations to provide feedback

Respondents varied in their views on minimum obligations to provide feedback. Eight viewed feedback as always obligatory while acknowledging practical barriers (i.e., factors that appear to be overwhelming obstacles), one even asserting that feedback would be unlikely without regulation. Two maintained that the obligation to provide feedback is context-dependent. Various respondents cited a stronger obligation with factors such as the severity of the potential harm, the likelihood of repeat errors, and the degree of certainty. Finally, 1 saw an obligation to provide feedback only if required for future care and another perceived no obligation (see Table 3 for illustrative quotes).

**Table 3:**
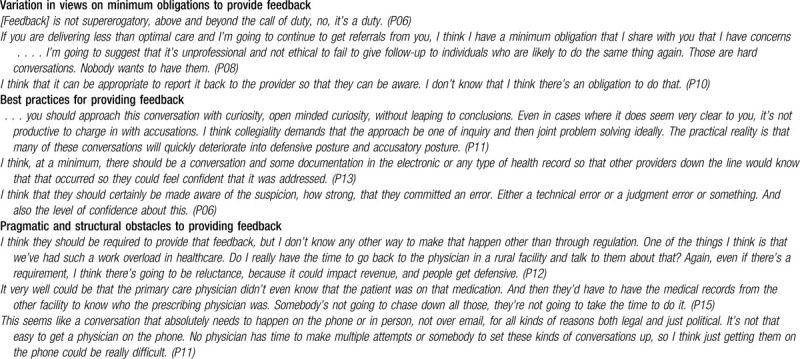
Best practices and minimum obligations for feedback to responsible providers about inter-system medical error discovery.

#### 3.2.2 Best practices for providing feedback

Five respondents advised providing feedback prior to taking any reporting action. Several advised training programs in relevant skills including communication and teamwork. Some noted structural barriers to feedback, such as the need for clarification of regulatory protections from discoverability, the need for changes to the healthcare system to counteract income dependency on referrals, and lack of time available for feedback conversations. Some favored systems permitting open communication between quality committees.

### 3.3 Reporting to external authorities

Respondents noted that reporting regulations vary by state and none were aware of requirements specific to IMED. Most respondents viewed reporting to the responsible provider's own system as akin to feedback and different than reporting to external bodies. Many noted that IMED errors must be more serious to reach an obligation to report compared to disclosure and feedback obligations. Seriousness was expressed in factors such as repetition of errors and risk to future patients. Other mediating factors included egregiousness, negligence, incompetence, uncertainty, severity, the type of error, the level of insight exhibited by the responsible provider, and the reporting body's capacity to do an appropriate, fair review (see Table 4 for illustrative quotes).

**Table 4:**
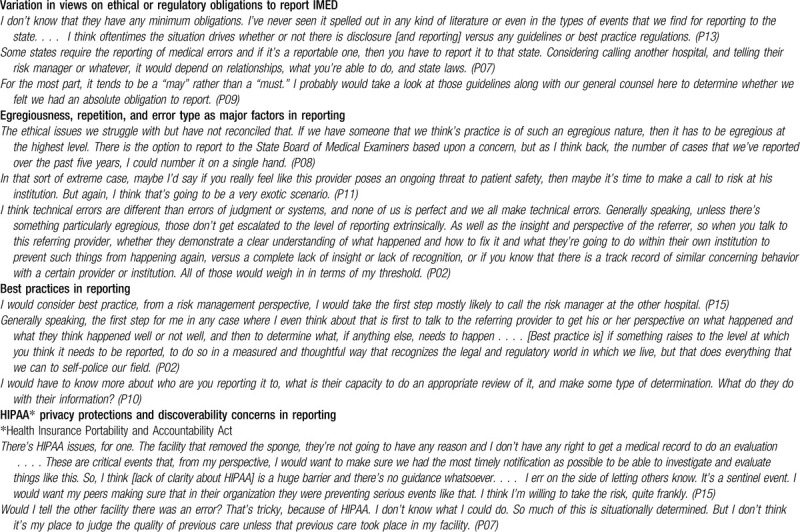
Best practices and minimum obligations for reporting IMED (inter-system medical error discovery) to the responsible provider's institution or external authorities.

### 3.4 Reasons for disclosure, feedback, or reporting

Respondents volunteered a variety of reasons justifying whether and how to take disclosure, feedback, or reporting actions in response to IMED (Table 5). Many reasons correspond with those identified in prior studies.^[6,8,21]^ While a universally cited reason for action was honesty and transparency, some interpreted transparency to mean communicating only what is known with certainty, whereas for others it meant communicating what is believed with clarity about the limits of the information. Some noted the need to weigh interactions between multiple factors before determining minimum obligations or best practice.

**Table 5:**
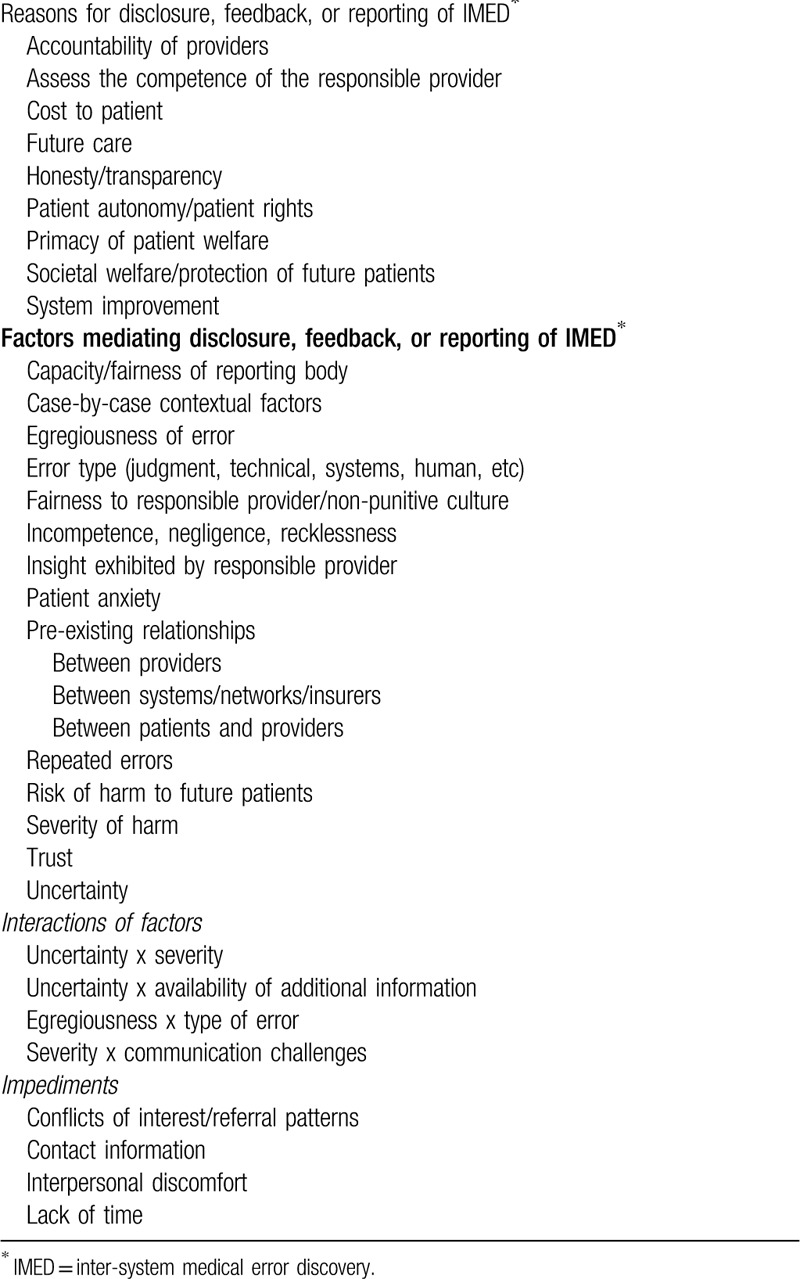
Reasons for actions and mediating factors.

### 3.5 Inter-system compared with intra-system errors

Four respondents reasoned that if the ethical standard for errors that occur within a system (intra-system errors) would require disclosure, then the same standard should apply to inter-system scenarios. One argued that nothing about the inter-system scenario per se could obviate the ethical obligation of disclosure to patients. Another extended this reasoning to reporting to external authorities, that is, if the guideline would require reporting of an error that occurred within one's system, the same guideline should be applied to those from another system (Table 6). Several ethicists raised concerns about unintended consequences, such as increasing patient distress, harm to institutional reputations, or reduction in access to healthcare, but allowed that these were secondary to ethical obligations for disclosure.

**Table 6:**
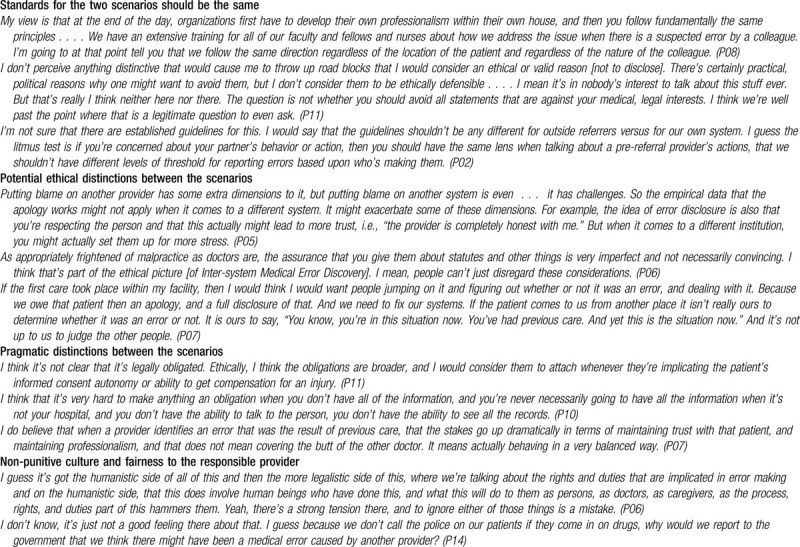
Inter-system medical error discovery scenarios in comparison to intra-system scenarios.

Many respondents discussed the pragmatic distinctions between the scenarios, some of which were reported previously.^[6,8]^ These include lack of clarity about legal issues such as Health Insurance Portability and Accountability Act (HIPAA) restrictions, protection of inter-system communications from discoverability, protection from liability when disclosure occurs, legal obligations to disclose, and liability for slander. In addition, challenges of communicating across systems, incomplete information, conflicts of interest due to referral patterns, and discomfort in conducting feedback conversations were cited, as well as an overall lack of time to address these obstacles.

Nearly all respondents discussed considerations of fairness to the responsible provider, some framing the discussion in terms of the need for a just and non-punitive culture in medicine. These concerns are not unique to inter-system medical errors but may be even more salient in a context of uncertainty about how multiple systems may respond. Concerns about excessively punitive reactions are reflected in many of the illustrative quotes (Tables 2–4) and in the negative metaphors used in relation to disclosure and reporting. Five respondents alluded to disclosure or reporting of responsible providers as “throwing someone under the bus,” 3 referred to reporting as “policing,” and 3 others advised against “finger pointing.” Four respondents made reference to the “punitive environment in healthcare” and the need to reframe feedback as motivated by patient safety (Table 6).

## 4 Discussion

The results of this exploratory study reveal that medical error professionals generally agreed on full, timely disclosure to patients of IMED when errors are clear-cut and harmful. This stance aligns with expectations of patients reported previously.^[21]^ However, specialists in a previous study differed in whether they would disclose IMED to patients^[6]^ and an analysis of relevant guidelines found variation among recommendations and a lack of specificity.^[9]^ While medical error professionals concurred about this obligation, they also noted practical and political barriers to IMED disclosure, as well as the lack of explicit guidance or systemic supports to facilitate disclosure.

In IMED cases with uncertainty or low severity of harm, professionals’ views diverged with some no longer perceiving a strong obligation to disclose. They also expressed varied opinions about best practices for disclosure. Many advised first investigating the error through discussion with the responsible provider in every case but differed in whether feedback should be obligatory.

Cases of medical error uncertainty have been identified as challenging but common, even when only one system is involved. Best practices proposed for medical error uncertainty emphasize handling disclosure as “a process, not an event”^[27]^ and understanding that uncertainty does not obviate the patient's expectation of transparency.^[21,27]^ We propose that these same ethical and practical principles for disclosure of errors with uncertainty should be extrapolated to IMED scenarios. What distinguishes IMED is that identifying appropriate contacts and fact finding across health systems is more difficult and time consuming than within a system. A full investigation is also hampered by lack of clarity about HIPAA restrictions and discoverability protections, while the motivation for self-improvement that prevails in internal root cause analysis is absent. Given these constraints, in addition to making a strong effort to investigate, the discovering system should give the patient enough information to pursue clarification of a potential error directly with the responsible system.

The divergence of opinions about disclosure of IMED with uncertainty may reflect an underlying lack of true consensus about medical error transparency. Despite best practice guidelines, in reality systems vary in disclosure of their own errors.^[28,29]^ For example, some may disclose that an “unanticipated clinical outcome” occurred without taking the next step of assessing whether the provider's actions were reasonable or erroneous under the circumstances. IMED amplifies the opportunity to sustain ambiguity in this manner.

Respondents’ concerns about whether the responsible provider would be treated fairly and non-punitively following disclosure or reporting raises the question of individual versus systemic responsibility for medical errors. Assertions that nearly all medical errors are owing to systemic, not human, problems do not seem supported by the data.^[30]^ As Sidney Dekker wrote, “At the sharp end, there is almost always a discretionary space into which no system improvement can completely reach. Systems cannot substitute the responsibility borne by individuals within that space.“^[31]^ Greater clarity about discovering providers’ ethical obligations may help improve transparency, accountability, and patient safety in such cases. At the same time, there is ample room for health systems to support providers throughout their response to IMED scenarios. The medical error professionals in this study pointed to important ancillary supports such as proactive or real-time communication training and support from error resolution teams. This aligns with Gallagher's suggestion that health systems use a “neutral third party” such as an ethics committee to make determinations about IMED responses in order to overcome provider biases or limitations.^[18]^

At the state and federal level, many respondents noted a lack of clear guidance on reporting IMED and confusion about regulations relevant to communication with other systems about errors. Despite Institute of Medicine recommendations, Congress has not legislated protection of information sharing between unaffiliated systems for purposes of error prevention.^[32]^ Patient Safety Organizations (PSOs), a program of the Agency for Healthcare Research and Quality, hold promise for planned sharing of de-identified patient safety data but are unlikely to resolve the difficult questions around legal discoverability and patient privacy in IMED scenarios. A state-by-state legal analysis of existing mandates and protections regarding IMED reporting and data sharing may help to resolve these uncertainties.

This study had several limitations. Due to the small sample size, results cannot be generalized to all medical professionals and comparisons between fields were not possible. Results may have been biased by respondent-driven sampling. However, in relation to the aim of the study, which was to explore consensus and divergence among medical error professionals, the sample was sufficient. These data, along with other preliminary data, will inform a future stakeholder consensus process to develop and test a toolkit for hospitals to use in responding to IMED. Our lack of initial knowledge about which factors would prove most pertinent resulted in some overly broad questions and ambiguous definitions. This is to be expected in an as yet unsettled ethical area. We addressed this limitation by improving the interview questions in subsequent interviews.

## 5 Conclusions

How providers and their health systems should respond after discovering a medical error that occurred in a different system remains an unsettled question. Professionals such as clinical ethicists, risk managers, medical attorneys, and patient safety experts represent an important stakeholder group with experience in medical error response. While medical error professionals agreed that clear-cut, harmful IMED errors must be disclosed to patients, they differed on best practices for IMED disclosure, when feedback to the responsible provider or reporting are required, and how obligations may change when mediating factors such as uncertainty or severity of harm are introduced. They also noted the lack of specific guidelines or clear regulations, with some asserting that this absence does not obviate the obligation to respond. Any future effort to develop clearer guidelines for responding to IMED must take into account these factors, as well as the practical and political barriers to communication about medical errors discovered across systems.

## Acknowledgments

Dr Dossett and this work is supported by a grant from the Agency for Healthcare Research and Quality (AHRQ; K08HS026030-01A1; PI-Dossett).

## Author contributions

JM and LD designed the study. JM acquired the data. JM and CAV analyzed the data. JM wrote the manuscript. RB and LD provided supervision and critical revisions. All authors contributed and approved of the final version.

## Supplementary Material

Supplemental Digital Content

## Supplementary Material

Supplemental Digital Content
