# Supplementary material for: Medical error professionals’ perspectives on Inter-system Medical Error Discovery (IMED): Consensus, divergence, and uncertainty
Source: Medicine (Baltimore). 2020 Jul 31;99(31):e21425. doi: 10.1097/MD.0000000000021425 (PMC7402729; doi:10.1097/MD.0000000000021425)
Supplement: Supplemental Digital Content [file medi-99-e21425-s001.docx]

**SUPPLEMENTARY MATERIAL A**

Sample interview guide
*Note: Guides were adapted to the expertise of the participant.*

**Interview Guide – Medical Ethicists**

1. Before we begin the interview itself, I’d like to confirm that you have read the informed consent form, that you understand that your participation in this study is entirely voluntary, that you may refuse to answer any questions, and that you may withdraw from the study at any time. Do you have any questions before we begin?
2. How would you describe your field? Do you have a sub-field relevant to medical errors? What is your current age, years of experience in your field, and years of experience in your sub-field, if applicable?
3. The Institute of Medicine defines medical errors as either a planned action improperly completed, or a plan that is inappropriate in the first place. A pre-referral medical error is an error discovered *after* a patient is referred to another healthcare professional. For example, a patient undergoes surgery for cancer. When the patient is later referred to a specialist for consultation, the second surgeon discovers that the original surgery was incomplete, necessitating a return to the operating room. That error, committed by the referring physician and discovered (in this example) by the specialist, is a pre-referral medical error.
4. Have you heard of cases where pre-referral medical errors were discovered by a later provider?
5. [If yes] Can you give an example?
6. [If yes] In this example, how did the pre-referral error come to light? Was the suspected error disclosed to the patient? By whom? Was it reported to any governing body? Was there communication between the referring provider and the downstream provider?
7. What do you think providers typically do when they discover a pre-referral medical error? Do they disclose the error to the patient?
8. Are you aware of any published ethical guidelines with respect to disclosure of pre-referral medical errors?
9. What do you consider ethical best practice in regard to disclosure of pre-referral medical errors to patients? Why?
10. What factors should be considered in determining whether a medical error should be disclosed?
11. Who do you think should make the determination about whether a pre-referral medical error has occurred?
12. What ethical obligations, if any, do providers have with respect to disclosure of pre-referral medical errors to patients after they discover them?
    1. First, consider a case where the pre-referral error is clear and unambiguous.
    2. Now consider a case where it is not entirely clear whether there a medical error was committed by a referring healthcare provider.
13. Can you think of any ethical guidelines, or other tools or mechanisms, that could make it easier for later providers to a) make determinations about disclosure of suspected pre-referral errors to patients and, b) if appropriate, disclose those errors?
14. Now we are going to move on to reporting of pre-referral errors. By reporting we mean to an organization, body or system other than the referring physician. Examples may include safety committees at the referring hospital, superiors or supervisors of the referring physician, hospital administrators, quality improvement systems, or credentialing or licensing boards. I am not talking about communication with the referring physician—we will label that “feedback.” Have you heard of situations where a pre-referral medical error was reported?
15. [If yes] Can you give an example? To whom was the error reported?
16. In your experience or estimation, do you think physicians typically report pre-referral medical errors when they discover them? Why or why not? If yes, to whom do you think they typically report?
17. What would you consider to be ethical best practice with regard to reporting of pre-referral errors by later providers? Why?
18. What potential ethical obligations, if any, do later providers have with respect to reporting of pre-referral errors?
19. Can you think of any ethical guidelines, or other tools or mechanisms, that could make it easier for later providers to report suspected pre-referral errors?
20. Let’s move on to feedback. By feedback we mean communication about pre-referral medical errors with the referring provider. What do you think physicians typically do with regard to feedback to referring providers if they discover a pre-referral error?
21. What potential ethical obligations, if any, do later providers have with respect to feedback?
22. Can you think of any ethical guidelines, or other tools or mechanisms, that could make it easier for later providers to give feedback to referring providers?
23. Anything else you would like to add?
