# Supplementary material for: Medical error professionals’ perspectives on Inter-system Medical Error Discovery (IMED): Consensus, divergence, and uncertainty
Source: Medicine (Baltimore). 2020 Jul 31;99(31):e21425. doi: 10.1097/MD.0000000000021425 (PMC7402729; doi:10.1097/MD.0000000000021425)
Supplement: Supplemental Digital Content [file medi-99-e21425-s002.docx]

**SUPPLEMENTARY MATERIAL B**

Codebook

| **Code** | **Definition** |
| --- | --- |
| **1 Good quotes** | Exemplary or interesting quotes |
| **2 Ambiguous definition-guidance** | Discussion of ambiguity about definitions and categories of provider-centered problems, and related ambiguity in guidance about what to do about those problems. For example, the overlapping constructs of medical error, adverse event, near miss, incompetence, impairment, unethical behavior, illegal behavior, negligence, malpractice, etc. If the ambiguity is about the clinical question of whether the error happened or who is responsible, instead code "Uncertainty." |
| **3 Ancillary supports** | Discussion of ancillary services that could support the IMED process, such as communication training, counseling services for providers and patients, legal advisement, etc. Consider co-coding with Ethical consultation and Risk management, as appropriate. |
| **4 Case examples** | Code when participant discusses specific cases, as in initial interview questions |
| **5 Ethical analysis** | Explicit discussion of ethical frameworks as applied to IMED errors, e.g., principle analysis, utilitarian analysis, etc. Also include uncertainty around ethical guidance. |
| **6 Ethics consultation** | Specific discussion of ethics consultations, staff, committees, roles, or processes |
| **7 Ethics v. practical/policy** | Explicit discussion of whether IMED is a policy or ethical issue, or both |
| **8 Investigation first** | Discussion of need to investigate IMED circumstances before disclosure, reporting, and/or feedback. Also consider "Fairness to responsible provider," "Uncertainty," and "Punitive culture." |
| **9 Legal issues** | Includes "sorry" laws, state or other mandates, medical liability caps, legal privileges, other state laws, legal guidance, legal obligations, HIPAA, etc.; co-code with Variability if applicable. Also code for mention of litigiousness in society. |
| **10 Metaphors** | Metaphors, similes, or other colorful language, e.g., "throw them under the bus" |
| **11 Non-explicit language** | Discussion of cloaking language that might be used when talking about an IMED error to a patient or to the responsible provider. For example, not using the word "error," saying "it's not what I would have done," "current health status," etc. (may or may not rise to the level of actual disclosure or feedback). |
| **12 Non-punitive culture** | General discussion of just culture, need for non-punitive culture, everyone makes mistakes, errors do not equal incompetence, etc. Consider more specific codes: "Fairness to responsible provider" and "Investigation first." |
| **13 Professionalism** | General invocation of professionalism or professional responsibility; includes self-regulation of profession and collegiality. Also consider "Fairness to responsible provider." |
| **14 Published guidelines** | Code for question regarding knowledge of any published guidelines on IMED error disclosure |
| **15 Risk management/QI** | Specific discussion of risk management or quality or safety offices, staff, roles, or processes |
| **16 Scenarios same or different** | Explicit discussion of differences, or lack thereof, between IMED errors and single system errors; include definitional issues |
| **17 Unable to answer** | Code when participant feels unable or unwilling to answer a question |
| **18 Unintended consequences** | Discussion of potential unintended consequences to disclosure, reporting, or feedback, such as no improvement in patient care or lack of benefit to error disclosure |
| **19 Variability** | Variability in practices, cultures, laws, or contexts between individuals, facilities, institutions, states, regions; co-code with Legal issues if applicable |
| **20 DRF Reasons** | Reasons given by participant for best practices around IMED disclosure or reporting |
| **20.1 Accountability** | Disclosure to ensure doctor's general accountability for errors and care. Also consider "Honesty-transparency." |
| **20.2 Assess competence** | Disclosure so the patient can assess doctor’s clinical competence and quality (distinct from ethical trust) |
| **20.3 Cost to patient** | Disclosure or reporting so patient can recover costs of care related to the error or consider legal options. |
| **20.4 Future care - reason** | Disclosure so patient can prepare for future care. Include discussion of disease progression and need for timely care. |
| **20.5 Honesty-transparency** | Disclosure because of values of honesty from a doctor, need for trust in a doctor’s ethics (as distinct from clinical trust), and/or transparency in communication with doctor |
| **20.6 Patient autonomy-rights** | Disclosure because of patient autonomy, e.g., "it's my body," "people should be able to choose for themselves," or patient’s rights in general |
| **20.7 Patient welfare first** | Disclosure because it's the doctor's obligation/duty to put the patient's interests ahead of any competing interests (if concern is for patient's psychological distress, code “DRF Factors\Patient anxiety” instead) |
| **20.8 Societal welfare** | Potential for harm to society or future patients as a reason for disclosure, reporting, or feedback. |
| **20.9 System improvement** | DRF for reasons of system improvement, even of harmless errors or near misses. |
| **21 DRF Factors** | Discussion of factors to be considered with respect to IMED error disclosure, feedback, or reporting. |
| **21.1 Context** | Discussion of context around IMED error as a factor to consider with respect to disclosure, reporting, or feedback, when specific context not otherwise specified. Consider more specific codes such as "Error type," "Future care," "Repeated errors," "Patient anxiety," "Severity," and "Uncertainty." |
| **21.2 Egregiousness** | Egregiousness of the error as a factor influencing disclosure, reporting, or feedback, i.e., should the doctor have known better, how "blatant" or negligent was it (also consider "Severity" which can be co-coded, but is distinct from the question of knowing better). |
| **21.3 Error type** | Discussion of different types or causes of errors, e.g., systems, human, judgement, technical, inattention to detail, mistake, failure to refer, etc. |
| **21.4 Fairness to responsible provider** | Concern that responsible provider will not be treated fairly after disclosure or reporting. Also consider "Investigate first," "Uncertainty," "Punitive culture," and "Patient welfare first." |
| **21.5 Interaction of factors** | Discussion of weighing multiple factors, interaction effects, benefit-harm assessment, gradient of obligation, etc. (e.g., even with uncertainty, the more significant the harm, the greater the obligation for disclosure). |
| **21.6 Patient anxiety** | Patient anxiety, mental health, or therapeutic privilege as a factor to consider in WHETHER to disclose (if only a factor in how or when to disclose, code “Error Disclosure\How” instead) |
| **21.7 Pre-existing relationship** | Discussion of impact of having a pre-existing relationship on issues of feedback, disclosure, or reporting. |
| **21.8 Repeated errors** | Repetition or pattern of errors as a factor to consider in disclosure, reporting, or feedback. |
| **21.9 Severity** | Severity of consequences as a factor to consider, ranging from any harm at all to treatability, irreversibility, disease progression, life-threatening, life-altering; include discussion of a "threshold" definition of error, minor errors, etc. |
| **21.10 Trust** | Discussions of factors related to trust in the healthcare system, trust between discovering provider and patient, trust between responsible provider and patient, trust between providers, or trust by providers in a reporting system. Also consider “Disclosure reasons/Honesty-Transparency.” |
| **21.11 Uncertainty** | Uncertainty about any circumstance around an IMED error as a factor mediating DRF; lack of access to information, grey areas, capacity to determine whether error occurred, uncertainty around standard of care, framing effects, etc. Also include differences of opinion in standard of care, situations of clinical equipoise, etc. Consider co-coding with "Investigate first." If the uncertainty is about ambiguous/nebulous/overlapping definitions of provider-centered problems (e.g., medical errors, adverse events, impairment, etc.), code "Ambiguous definition" instead. |
| **21.12 Who determines error** | Code for questions (potentially under 1) disclosure section and 2) closing section of interviews) about who should determine whether an error has occurred and by whom; consider co-coding with "Uncertainty" and "Investigate first." |
| **22 Impediments** | Discussion of impediments, barriers, or constraints to disclosure, reporting, and/or feedback. |
| **22.1 Conflicts of interest** | Discussion of conflicts of interest as a barrier, including referral base, financial competition, financial dependence, reputational fears, etc. |
| **22.2 Contact information** | Lack of clarity about who to contact or how as a barrier to reporting or feedback about IMED errors. |
| **22.3 Interpersonal discomfort** | Encompasses unpleasantness of having disclosure conversation with patient, fear of conflict with other providers, fear of appearing judgmental/arrogant, etc. |
| **22.4 Lack of time** | Lack of time as an impediment to disclosure, reporting, or feedback. |
| **23 Error Disclosure** | Disclosure of medical errors. |
| **23.1 Disclosure tools-solutions** | Discussion of tools, guidelines, mechanisms, or other potential solutions related to disclosure. |
| **23.2 How to disclose** | All discussion of when and how to disclose, and by whom; include discussion of urgency, multiple stages of disclosure, investigation first, etc.; can co-code with factors if applicable |
| **23.3 Disclosure opt out** | Discussion of giving patients the option NOT to hear information about a medical error. Co-code with "Patient autonomy." |
| **23.4 Intra-system Disclosure** | Disclosure of one's own medical errors or those of others within one's own system. |
| **23.5 IMED Disclosure** | Disclosure of medical errors that occurred in another system only. For errors committed by someone else within one's own system, code “Intra-system disclosure.” |
| **23.5.1 Typical Disclosure** | Discussion of what discovering providers typically or currently do with regard to IMED error disclosure; include factors affecting this (e.g., career stage) if not elsewhere captured |
| **23.5.2 Best Disclosure** | Discussion of best practice with regard to IMED error disclosure |
| **23.5.3 Minimum Disclosure Duty** | Discussion of the minimum obligation of discovering providers with respect to IMED error disclosure |
| **24 Error Reporting** | Reporting to an organization, body or system other than the responsible physician. Include anonymous or confidential reporting systems. Exclude feedback to the responsible provider. |
| **24.1 Reporting process** | Code for any reporting process issues, such as uncertainty about where to report, what may happen after reporting, etc. Also consider "Contact information" and "Best Reporting Practice," if applicable. |
| **24.1.1 Anonymity** | Discussion of anonymity in reporting or anonymous and/or de-identified reporting systems. |
| **24.1.2 Formal v. informal reporting** | Discussion of informal reporting between facilities or systems and formal reporting to external bodies. |
| **24.2 Reporting tools-solutions** | Discussions of tools, guidelines, mechanisms, or potential solutions to reporting. |
| **24.3 Intra-system Reporting** | Reporting of errors discovered internal to a facility or system. |
| **24.4 IMED Reporting** | Reporting of medical errors discovered in another facility or system. |
| **24.4.1 Typical Reporting Practices** | Discussion of what discovering providers typically or currently do with regard to IMED error reporting; include factors affecting this (e.g., career stage) if not elsewhere captured |
| **24.4.2 Best Reporting Practice** | Discussion of best practice with regard to IMED error reporting |
| **24.4.3 Minimum Reporting Duty** | Discussion of the minimum obligation of discovering providers with respect to error reporting |
| **25 Error Feedback** | Communication between the discovering provider and the responsible provider about the error. |
| **25.1 Formal v. informal feedback** | Explicit discussion of formal versus informal mechanisms of feedback, such as written letter or record versus telephone. |
| **25.2 Feedback tools-solutions** | Discussion of tools, guidelines, mechanisms, or other potential solutions with regard to feedback. |
| **25.3 Typical Feedback Practices** | Discussion of what discovering providers typically or currently do with regard to IMED error feedback; include factors affecting this (e.g., career stage) if not elsewhere captured |
| **25.4 Best Feedback Practice** | Discussion of best practice with regard to IMED error feedback |
| **25.5 Minimum Feedback Duty** | Discussion of the minimum obligation of discovering providers with respect to IMED error feedback |
| **25.6 Indirect Feedback** | Indirect communication attempting to improve community care around medical errors or patient safety. Examples include peer review, mentoring, conferences, community education, etc. |
